# Generation and Initial Characterization of FDD Knock In Mice

**DOI:** 10.1371/journal.pone.0007900

**Published:** 2009-11-18

**Authors:** Luca Giliberto, Shuji Matsuda, Ruben Vidal, Luciano D'Adamio

**Affiliations:** 1 Department of Microbiology and Immunology, Albert Einstein College of Medicine, Bronx, New York, United States of America; 2 Department of Pathology and Laboratory Medicine, Indiana Alzheimer Disease Center, Indiana University School of Medicine, Indianapolis, Indiana, United States of America; Columbia University, United States of America

## Abstract

**Background:**

Mutations in the integral membrane protein 2B [1], also known as BRI_2_ [2], a type II trans-membrane domain protein cause two autosomal dominant neurodegenerative diseases, Familial British and Danish Dementia [3]. In these conditions, accumulation of a C-terminal peptide (ABri and ADan) cleaved off from the mutated precursor protein by the pro-protein convertase furin [4], leads to amyloid deposition in the walls of blood vessels and parenchyma of the brain. Recent advances in the understanding of the generation of amyloid in Alzheimer's disease has lead to the finding that BRI_2_ interacts with the Amyloid Precursor Protein (APP), decreasing the efficiency of APP processing to generate Aβ [5], [6], [7]. The interaction between the two precursors, APP and BRI_2_, and possibly between Aβ and ABri or ADan, could be important in influencing the rate of amyloid production or the tendency of these peptides to aggregate.

**Methodology/Principal Findings:**

We have generated the first *BRI_2_* Danish Knock-In (FDD_KI_) murine model of FDD, expressing the pathogenic decamer duplication in exon 6 of the *BRI_2_* gene. FDD_KI_ mice do not show any evident abnormal phenotype, with normal brain histology and no detectable amyloid deposition in blood vessel walls or parenchyma.

**Conclusions/Significance:**

This new murine mouse model will be important to further understand the interaction between APP and BRI_2_, and to provide insights into the molecular basis of FDD.

## Introduction

BRI_2_ is a type II trans-membrane protein of unknown function. The *BRI* gene belongs to a multigene family comprising at least three homologues in both mice and humans, *BRI_1_*, *BRI_2_* and *BRI_3_* (also referred to as *ITM2A*, *ITM2B* and *ITM2C*, or *E25A*, *E25B* and *E25C*, respectively) [1], [2], [3], [8], [9]. It possesses a BRICHOS domain, a conserved motif common to members of the BRI, ChM-I, SP-C and CA11 protein families, thought to have a role in the targeting of the respective proteins to the secretory pathway or to intracellular processing [10]. Proteins sharing the BRICHOS motif are dissimilar, and associate with a diverse range of phenotypes, varying from dementia to cancer and respiratory distress.

*BRI_2_* was first described in relation to Familial British Dementia (FBD) [2], an autosomal dominant neurodegenerative disease characterized by the early onset of personality changes, memory and cognitive deficits, spastic rigidity and ataxia. In FBD, a C-terminal 34 amino acid (aa) peptide of BRI_2_ accumulates as amyloid, leading to severe amyloid angiopathy of the brain and spinal cord with perivascular amyloid plaque formation, parenchymal plaques affecting the limbic areas, cerebellum, cerebral cortex, neurofibrillary tangles of hippocampal neurons and periventricular white matter changes [11]. Familial Danish Dementia (FDD), previously known as heredopathia ophthalmo-otoencephalica, is an autosomal dominant disease characterized by the accumulation of an amyloidogenic C-terminal 34 aa peptide of BRI_2_ [3]. FDD is characterized by early onset cataracts, deafness, progressive ataxia and dementia [3], [12]. Neuropathological examination of patients with FDD shows diffuse brain atrophy with a particularly severe involvement of the cerebellum, cerebral cortex and white matter, as well as the presence of very thin and almost demyelinated cranial nerves, and widespread amyloid angiopathy in the small blood vessels and capillaries of the cerebrum, choroid plexus, cerebellum, spinal cord and retina. In FDD, parenchymal compact plaques are consistently absent, whereas neurofibrillary tangles (NFTs) are the major histological finding in the hippocampus [3], [12].

BRI_2_ is physiologically cleaved, at the C-terminus, by furin, a calcium-dependent serine endoprotease, producing a 23 aa soluble C-terminal fragment. FBD and FDD are due to mutations in the *BRI_2_* gene located on chromosome 13q14 [2], [8]. In FBD, a single base substitution at the stop codon of *BRI_2_* generates a longer open reading frame, resulting in a larger, 277 aa precursor (BRI_2-ABri_, compared to the 266 aa long normal protein) [2]. In FDD, a decamer duplication in the 3′ region of the *BRI_2_* gene, right before the stop codon, leads as well to the production of a longer, 277 aa protein (BRI_2-ADan_) [3]. The genetic defect is different, but the outcome is the same: the generation of a longer 34 aa C-terminal fragment, ABri in FBD and ADan in FDD, which accumulates as amyloid [2].

Several neuropathological features are common to FBD, FDD and Alzheimer's disease (AD): amyloid deposition and neurodegeneration in the central nervous system (CNS), accumulation of complement proteins and their pro-inflammatory activation products, including iC3b, C4d, Bb, and C5b-9, neurofibrillary pathology and hyperphosphorylated tau [12], [13], [14]. Furthermore, in FDD, Alzheimer's Aβ co-deposits with ADan, mainly in vascular and perivascular amyloid lesions and less in parenchymal preamyloid deposits [12], while compact plaques are not frequent [2], [12], [15].

More recently, a new link between BRI_2_ and Aβ has been found. It has been shown that BRI_2_ binds APP in a region comprising the extracellular juxtamembrane domains of both proteins, in a cis fashion [5], [6], [7]. This interaction leads to interference on the physiological processing of APP: BRI_2_ restricts the docking of γ-secretase to APP and the access of α- and β-secretases to their cleavage site on APP itself. The overall result of this interaction is the reduction of the amyloidogenic processing of APP, without a direct inhibition of the general activity of the secretases [5]. BRI_2_ maturation is required for this function. In fact, only mBRI_2_ (and not the immature precursor) binds mature APP and inhibits its processing on the plasma membrane and in endocytic compartments [16]. Matsuda et al have further shown that BRI_3_, a member of the BRI family, binds and inhibit the action of α- and β-secretases on APP [17]. Others have argued that the BRI_2_-23 wild type C-terminal peptide, released from BRI_2_ by furin/furin-like cleavage, can inhibit Aβ aggregation in vitro [18].

The interaction between these two amyloidogenic proteins can be of interest, especially for the design of new therapeutic strategies for AD and FBD/FDD. To this end, the processing of the BRI_2_ precursor, both in the wild type allele and its mutant form must be understood. Transgenic mice bearing the British or Danish mutations have been generated, and are under investigation as models of dementias and cerebral amyloid angiopathies [19], [20]. A transgenic FDD model which over-expresses the Danish mutant form of BRI_2_ shows amyloid deposition in the walls of blood vessels of the cerebrum and cerebellum, parenchymal amyloid deposition and reactive gliosis, ADan amyloidosis and some signs of cerebellar ataxia [20]. Transgenic over-expression of human mutant genes has been extensively used to generate mouse models for human neurodegenerative disorders due to the need of over-expressing enough amount of the mutant protein in order to observe a phenotype. It is however possible that some disorders may, at least partially, be due to loss of function rather than a gain of toxic function due to mutations. If this were the case, a transgenic approach would be counterproductive. Therefore, we have reasoned that models reproducing the genetics of human pathologies may be worth studying since they may unveil important disease mechanisms that a transgenic approach may miss or, possibly, hide. Thus, we have generated a genetically faithful model of FDD by introducing the pathogenic human mutation in the mouse BRI_2_ gene by a knock-in technology. Here, we describe the generation of this Knock-In murine model of FDD (FDD_KI_) and its initial characterization.

## Materials and Methods

### Ethics Statement

Mice were maintained on a C57BL/6 background for several generations (at least 15). Mice were handled according to the Ethical Guidelines for Treatment of Laboratory Animals of Albert Einstein College of Medicine. The procedures were described and approved in animal protocol number 20040707.

### FDD_KI_ Mice Construction

To clone the ADan mutation allele, a fragment obtained form BAC (containing mouse BRI_2_ genome) was amplified by PCR with the following primers:

Fw: 5′-cccAAGCTTtttttttttttttttaaagacaac-3′; Rev: 5′-gggAAGCTTgaagtggtcagcagggag-3′, obtaining a 7536 bp fragment, flanked by 2 *HindIII* sites, and comprising part of Intron 2 to a 3′UTR region, up to 3850 bp from the BRI_2_ stop codon. Such a fragment was cloned into a pBS vector, into *HindIII* sites (pBS-Ex3-6 *HindIII*), and used as a *template* for subsequent cloning.

A 340 bp fragment containing the ADan (duplication 786–795 of cDNA) mutation, plus a humanizing substitution a→g (acc→gcc = Threonine→Alanine) at the 12^th^ codon of exon 6, was obtained by serial PCRs, using pBS-Ex3-6 *HindIII* as a template. The final external primers contained restriction sites for *HincII* (5′) and *EcoRI* (3′). The mutated fragment (*HincII*-ADan-*EcoRI*) was inserted into the *SmaI* and *EcoRI* sites of a pBS vector yielding pBS-ADan0.3.

Subsequently, 2 *EcoRI-EcoRI* and *EcoRI-XhoI* fragments (2.6 kbp total) were cut from the template and added 3′, in tandem, to pBS-ADan0.3, generating pBS-ADan2.9, containing the Right Arm (RA) fragment of the construct.

A *NotI-XhoI* fragment from pBS-ADan2.9, comprising the RA, was inserted into *NotI* and *SacII* sites of a Soriano PGK-Neo-dTA vector (blunt-ended by the *SacII* and *XhoI* digestion followed by treatment of T4-exonuclease and Klenow polymerase respectively) at 5′of the Neo cassette. The *XhoI* site was reconstituted upon ligation of the fragment into the vector.

The construct's Left Arm was extracted from the template with *EcoRI* and *HincII*, generating a 1.1 kbp into Intron 5 up to the corresponding *HincII* site, 5′ of Exon 6 (start of Right Arm). The fragment was thus inserted into the PGK-Neo-dTA vector at 3′ of the Neo cassette and 5′ of the dTA cassette.

The resulting construct was thus:

---Right Arm-LoxP1-PGK-Neomycin cassette-LoxP2-Left Arm-dTA cassette---.

The resulting construct was linearized with *SalI* and purified prior to injection in ES cells strain 129 by electroporation. ES culture was performed on feeder layer, and further electroporation and handling was also performed according to the methodology employed at Dept of Cell Biology, Albert Einstein College of Medicine, and according to Wakayama et al. [21]. In particular, after electroporation, ES cells were re-plated in 55 cm^2^ dishes and let grow until visible clones would appear. Clones were then picked and transferred to 96 well plates in triplicates. Triplicates were either screened by PCR or frozen for subsequent use and further analysis.

Homologous recombinants were selected with G418 (200 µg/ml) and dTA exclusion.

Injection of the two Danish ES cell clones into C57BL/6J blastocysts was performed at the Albert Einstein College of Medicine gene-targeting facility, according to the current facility protocol (http://www.aecom.yu.edu/home/SharedFacilities/ViewFacility.asp?ID=30).

### PCR Analysis

The PCR screening was performed using the Expand Long Template PCR System (Roche-applied-Science) with Betaine, according to the manufacturer instructions. PCR analysis of recombinant ES cells and mice was conducted with the following primers and digestion strategies to identify the correct recombinant clones and strains:

*ES cells:*

Left arm:

Genomic primer: 5′-CAGTCCTACCTTATCCATGAGCAC-3′

Neo cassette primer: 5′- CTTCCTCGTGCTTTACGGTATC-3′

Product: 1637 bp

Right arm:

Genomic primer: 5′- AGTCTGTATCTCACTACGGCATCC-3′

Neo cassette primer: 5′- TGCACGAGACTAGTGAGACGTG-3′

Product: 3403 bp

*Amplification of the inserted construct and digestion:*

Genomic upstream primer: 5′-CAGTCCTACCTTATCCATGAGCAC-3′

Genomic downstream primer: 5′-AGTCTGTATCTCACTACGGCATCC-3′

Product: 4300 bp = construct without Neo cassette; NotI digestion→2900 bp + 1300 bp

Product: 6109 bp = construct with Neo cassette; NotI digestion→3100 bp + 2900 bp

*Mice:*

Whole construct

*Set 1*

Fw primer: 5′-ATGGCACCACCCACAATAGG-3′

Rev primer: 5′-CCTAGCAACTGGTAACAGTGC-3′

Product: 2027 bp with Neo cassette

Product: 332 bp without Neo cassette

Product: 194 bp wild type

*Set2*

Fw primer: 5′-ATGGCACCACCCACAATAGG-3′

Rev primer: 5′-ggtaagctctaaggagaagcg-3′

Product: 2500 bp with Neo cassette

Product: 849 bp without Neo cassette

Product: 704 bp wild type

Due to the specific sequences present in the amplified PCR product, no digestion of the amplicon could be performed on the mice PCR analysis. PCR products were thus sequenced to ascertain that the targeted sequence was correctly inserted in the genomic DNA. The sequence has been deposited in GenBank (accession number GQ424832).

### Southern Blot Analysis

Twenty µg of genomic DNA was digested with *BamHI* overnight, run on a 1% TAE agarose gel and transferred on a Hybond-N+ membrane (Amersham).

The probe was prepared by PCR from a BAC clone (RP24) with the following primers:

-Left arm:

Fw: 5′- GACAGAGGTTCTGCCCTCAG-3′

Rev: 5′-ACCGAGTCGTAGGACAGTG-3′

Probe size: 547 bp

-Right arm:

Fw: 5′- CTGTGCTGCCTGACACTACTTC-3′

Rev: 5′- TCTGTCCATACTCCCTGTCCTT-3′

Probe size: 515 bp.

One µg of PCR probe was labelled with 5 µL of ^32^P-dCTP (3000 Ci/mmol, ICN) and purified through a Push Column (Stratagene) according to the manufacturer's protocol. Membranes, containing the cleaved genomic DNA, were hybridized at 65°C and subsequently washed 4 times in SSC buffer (Sigma). Film was exposed to the hybridised membranes at –80°C and then developed.

### General Pathology Analysis

Wild-type and FDD_KI_ animals were studied at 18 months of age. After anesthesia, animals were perfusion-fixed with 4% paraformaldehyde in 0.1 M phosphate buffer (pH 7.2) (Sigma), after which brains and organs were removed, embedded in paraffin and sectioned. Eight µm sections were cut and mounted on poly-l-lysine-coated slides. After deparaffinization in Xylene ad rehydration, sections were stained with hematoxylin and eosin (H&E), Congo red standard methods. Mineralization was visualized in H&E sections as a deep basophilic amorphous and/or granular material.

### Brain Histology and Immunohistochemistry

Wild-type, FDD_KI_ and FDD-Tg animals were studied at 18 months of age. After anesthesia, animals were perfusion-fixed with 4% paraformaldehyde in 0.1 M phosphate buffer (pH 7.2) (Sigma), after which brains were removed, embedded in paraffin and sectioned. Eight µm sections were cut and mounted on poly-l-lysine-coated slides. Sections were stained with H&E, Congo red and Thioflavin S (ThS) methods. Immunohistochemical stainings were carried out as described [20] with following primary antibodies: glial fibrillary acidic protein (GFAP) (Dako, Carpinteria, CA); ubiquitin (Dako); amyloid β protein (Aβ) clone 4G8 (SIGNET, Dedham, MA); and ADan amyloid peptide (Ab 1700) [20]. Immunostaining was visualized using the avidin-biotin system (Vectastain; Vector Laboratories, Burlingame, CA) and 3,3′-diaminobenzidine (Sigma) as the chromogen. The sections were counterstained with cresyl violet or H&E. ADan transgenic (FDD-Tg) mice were described elsewhere [20].

### Neuronal Cultures

Neuronal cultures of FDD_KI_ mice, and wt littermates, were performed as described previously [22], [23] from E16-17 fetuses.

### Western Blot

Western blot was conducted on protein extracts from brains or neuronal culture of FDD_KI_ mice and wt littermates, as described previously [5] for cells lysates and brain tissue. Briefly, primary neuronal cells were lysed in Hepes-Triton buffer (20 mM Hepes/NaOH pH 7.4, 1 mM EDTA, 150 mM NaCl, 0.5% Triton X-100, plus protease inhibitors (PIs)) on ice for 30 min. The lysates were cleared by spinning at 20,000 g for 10 min. Equal amount of proteins of cleared lysates were loaded on SDS-PAGE and transfered onto nitrocellulose membranes; APP and BRI_2_ (wild type and Danish mutants) were detected by 22C11 (Chemicon, MAB348) or BRI_2_ antibody, generously provided by Dr. Haruhiko Akiyama, respectively [24].

## Results

### Generation and Characterization of FDD_KI_ Mice

**Figure 1:**
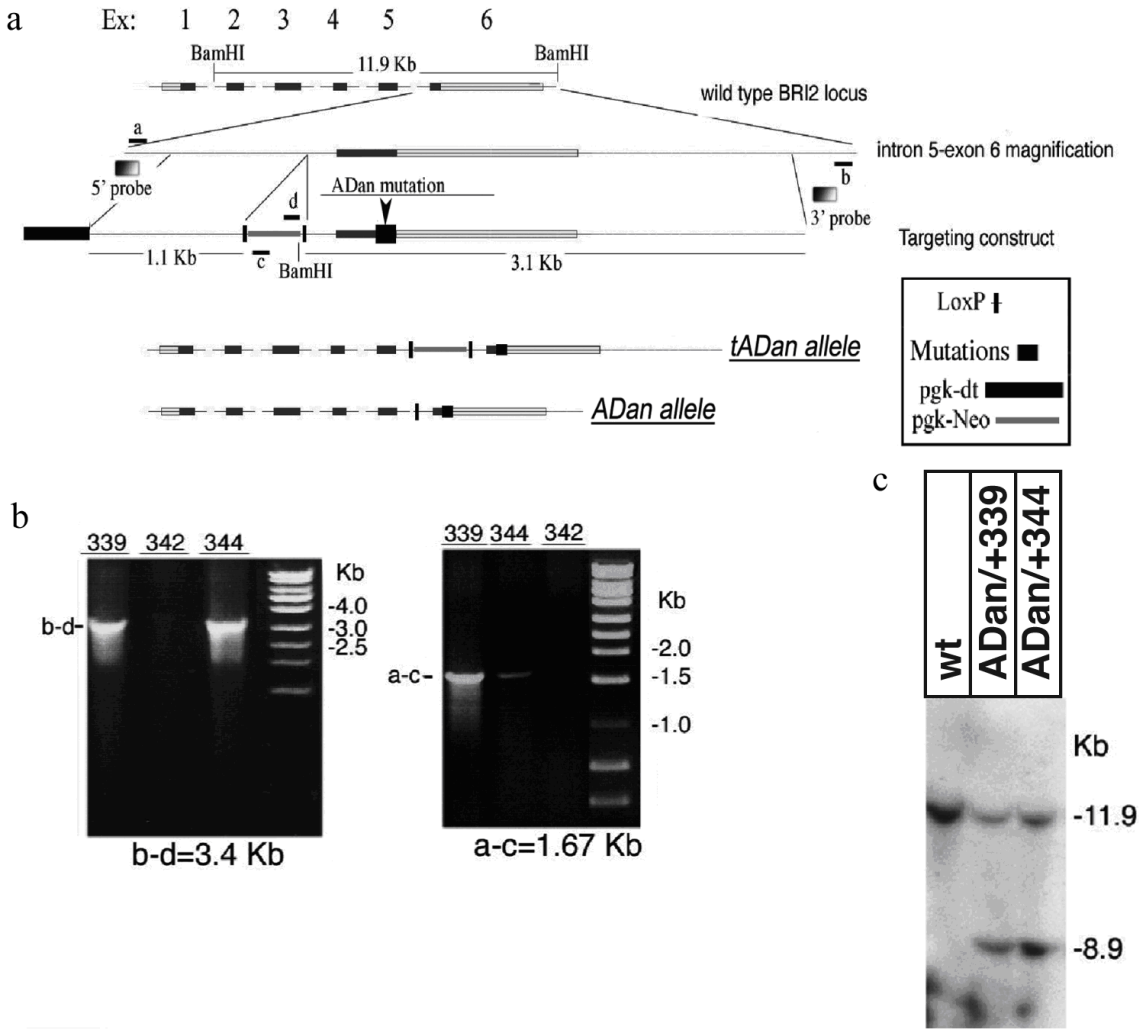
Generation of FDD_KI_ mice. 1a. Schematic representation of the construct that was injected in 129 ES cells, showing site of ADan mutation on Exon 6, primer sites, site of Southern Blot probe, LoxP, pgk-dt and pgk-Neo sites. The bottom graphics depict the construct with and without the pgk-Neo cassette that has been removed by means of Cre recombinase. 1b. Examples of 2 ES clones positive for the homologous recombination of the mutated allele: left arm (a–c: 1.67 kb) and right arm (b–d: 3.4 kb) PCRs are shown. 1c. Southern Blot showing a shift from the 11.9 kb of the wild type genome to the 8.9 kb band of the FDD KI mice, due to the insertion of a new *BamHI* site. KI mice show both bands, indicating heterozygosity.

The targeting strategy for the generation of the Danish mutant FDD_KI_ mice entailed the replacement of the *BRI_2_* exon 6 with a mutated exon 6 carrying the FDD mutation. We generated a targeting vector for the introduction of FDD BRI_2_ mutation. The vector used the floxed *PGK-neo* selection cassette and contains a 5′ homologous region and the negative selection cassette, *PGK-dt*. The 3′ homologous region introduced the FDD mutation and a *BamHI* site into the BRI_2_ mouse gene. The 10 nucleotide insertion found in patients with FDD was introduced before the stop codon. In order to humanize the mouse C-terminal region of BRI_2_, an alanine (A) was substituted for threonine (T) at codon 250 (Fig. 1a) of the murine *BRI_2_* gene.

The linearized targeting vector was transfected into 129 ES cells by electroporation. In the presence of the positive selection drug, G418, only those clones in which the *PGK-neo* selection cassette was integrated and the *PGK-dt* cassette was removed by homologous recombination would survive. ES cell clones carrying the targeting vector by random, non-homologous integration, were eliminated due to expression of diphtheria toxin.

After selection in G418-containing medium, ES cell clones carrying the proper homologous recombination and the *tADan* (targeted ADan) allele were identified by PCR for 5′ region (i.e. Left Arm: if homologous recombination had occurred these primers would amplify a product of 1.67 kb) and for the 3′ region (i.e. Right Arm: if homologous recombination had occurred these primers would amplify a product of 3.4 kb). Out of the ∼600 screened ES clones, we found three clones in which the Danish mutation was inserted in one of the *BRI_2_* alleles. Representative positive (BRI_2_ *^tADan/+^*344 and BRI_2_ *^tADan/+^*339) and negative (BRI_2_ *^tADan/+^*342) ES clones are shown in Fig. 1b. Also, PCR amplification and digestion, as specified in the Material and Methods section, was used to check the proper insertion of the construct in the genomic DNA and the removal of the Neo cassette (not shown).

The occurrence of homologous recombination was confirmed by sequencing the PCR products and by performing Southern blot analysis (Fig. 1c). DNA derived from individual BRI_2_ *^tADan/+^* ES clones was digested with *BamHI*, gel separated, blotted into a nylon membrane and hybridized with either the 5′ or the 3′probe. The 5′ probe hybridizes with a ∼11.9 kb fragment derived from the wild-type locus. Homologous recombination at the 5′ homologous region yields a ∼8.9 kb fragment upon *BamHI* digestion due to the introduction of the *BamHI* site and the *PGK-neo* selection cassette. ES clones BRI_2_ *^ADan/+^*344 and BRI_2_ *^ADan/+^*339 carry a wild type allele (11.9 kb) and a recombined allele (8.9 kb). Of note, the 11.9 kb and 8.9 kb bands had a similar intensity, indicating that 50% of the BRI_2_ alleles are wild type and 50% are recombined, and proving that the ES cells we have selected are a clonal populations. Similar results were obtained when homologous recombination at the 3′ site was assessed. In this case the 3′ probe detected a wild-type ∼11.9 kb fragment and a recombinant 4.7 kb fragment, due to the introduction of the *BamHI* site.

These two Danish ES cell clones (129, agouti coat colour), carrying the correct site-specific homologous recombination, were injected into C57BL/6J blastocysts (black coat colour) at the Albert Einstein College of Medicine gene-targeting facility. The resulting chimeras with a high proportion of agouti coat colour (*i.e.* with a high relative contribution from the injected ES cells) were backcrossed to C57BL/6J mice to obtain heterozygous BRI_2_ *^ADan/wt^*, which were identified by PCR and Southern analysis as described above (not shown) using tail DNA. Heterozygous mice were crossed to Meu40-Cre mice to obtain Meu40/BRI_2_ *^tADan/wt^* animals. Cre is a bacteriophage P1-encoded recombinase that catalyzes site-specific recombination between two 34 bp loxP recognition sites, resulting in the excision of the intervening DNA sequences. The resulting mouse has been named FDD_KI_.

### General Characteristics and Pathology of the FDD_KI_ Mice

Mice presented with no growth abnormalities, thrived at appropriate age, as their wild type littermates. Up to age 18 months, no susceptibility to infections was noted. The animals are active and alert.

Before the pathology examination, at 18 months of age, they presented in good body condition with adequate body fat, with no discharges or secretions from nostrils, conjunctiva, aural, urogenital or anal openings. Some mice also presented heart valve melanosis, which is a common finding in aged mice of various strains. Other sporadic findings, which are common in aged mice, were small foci of hepatocellular necrosis, vacuolation of and degeneration/regeneration of kidney tubular epithelial cells, mineralization in the kidney tubules in the pelvis and cortico-medullary junction (not shown). Overall, these mice have lesions that are commonly found in older mice and are considered age-related, spontaneous lesions. All of the lesions found were considered to be within the normal limits for age-related lesions. One of the animals presented with a uterine histiocytic sarcoma and hydronephrosis, both of which are found in older, untreated mice at low incidence.

### Neuropathology

**Figure 2:**
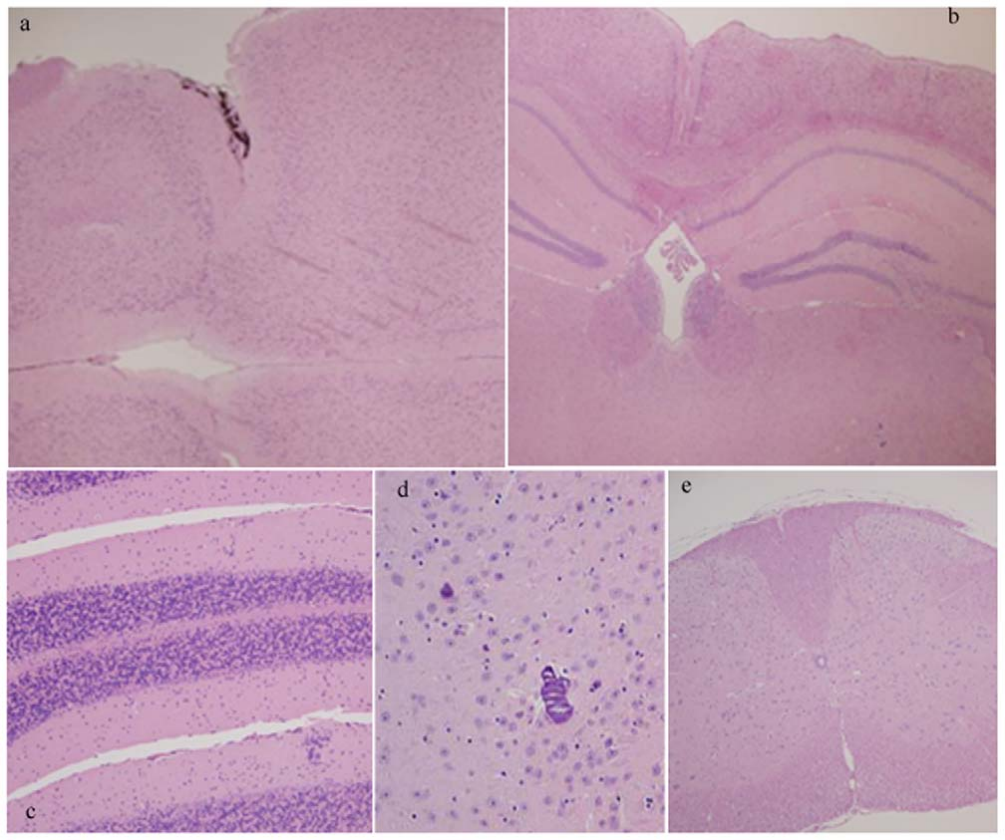
General Neuropatholgy. H&E staining of 18 month old FDD_KI_ mice showing signs of melanosis in the meninges of olfactory bulb (a) and signs of mineralization in the thalamus (d). The remaining parenchyma of brain cortex and hippocampus (b), cerebellum (c) and spinal cord (e) show normal cellular structures.

A few animals presented a small amount of dark basophilic material deposited on either side of the lateral thalamus consistent with mineralization (incidental, Fig. 2d), which is a common finding in aged mice of various strains, and the deposits develop along the basement membranes of the vasculature and may contain calcium and phosphorus. The finding of dark brown pigment along the meninges of the olfactory bulbs is consistent with melanosis (incidental, Fig. 2a), which is also a common finding in mice in dark pigment mouse strains such as C57BL/6 or having that strain the their background. All the brain tissue and the spinal cord were stained with Congo red for potential amyloid deposition. All of the stained tissue was negative for amyloid (not shown). Samples of H&E staining of cerebral cortex, hippocampus, cerebellum, thalamus and spinal cord are shown in Fig. 2a–e.

**Figure 3:**
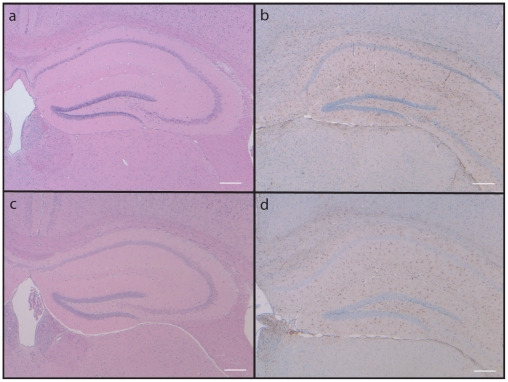
Sections of the hippocampus of FDD_KI_ mice. Sections from a wild type (a, b) and a homozygous knock-in mouse (c, d) were stained with H&E (a, c). Sections were also studied by immunohistochemistry using anti-GFAP (b, d). No significant differences were observed between wild-type and Knock In mice. Scale bars: a–d, 200 µm.

**Figure 4:**
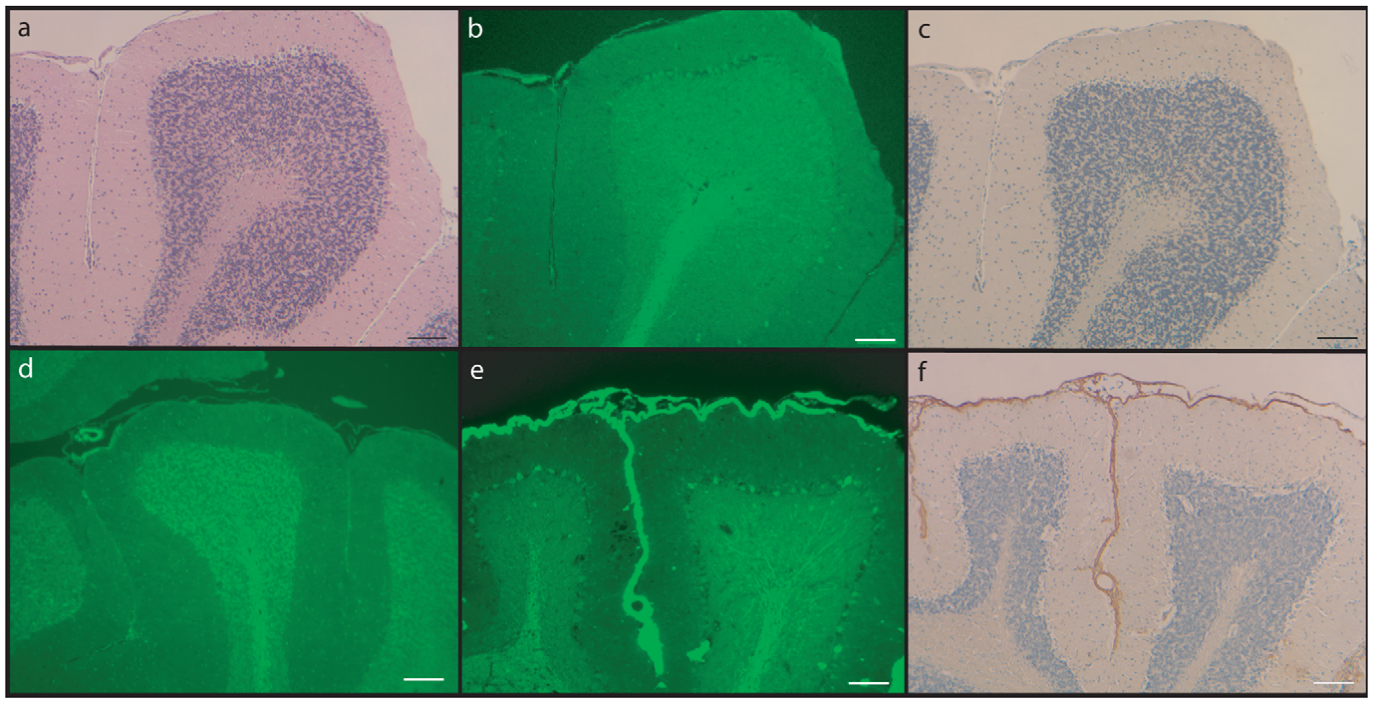
Sections of the cerebellum of FDD_KI_ mice. Sections from a wild-type (d), homozygous knock-in mouse (a–c), and FDD-Tg mouse (e, f) were stained with H&E (a) and ThS (b, d, e). Sections were also studied by immunohistochemistry using Ab 1700, specific for the ADan amyloid peptide (c, f). No significant differences were observed between wild-type and knock-in mice. Staining with ThS and Ab 1700 shows the presence of ADan amyloid only in FDD-Tg mice (e–f). Scale bars: a–e, 100 µm; f, 200 µm.

Brain sections of 18-month-old mice were examined for FDD-related pathology, particularly for amyloid deposition in brain parenchyma and vessel walls. For comparison, we included sections of an age-matched female FDD-Tg mouse, which expresses the Danish mutant form of human *BRI_2_* under the mouse prion protein promoter [20]. H&E staining of Knock In mice (Fig. 3c, 4a) showed no significant loss of neurons or noticeable malformations, and this observation was further confirmed when mutant sections were compared with those of wild type littermates (Fig. 3a, 4d). Immunostaining using anti-GFAP (Fig. 3b, d) and anti-ubiquitin (not shown) antibodies did not reveal significant differences with wild type mice in levels of activated astrocytes or presence of ubiquitinated material, respectively. Staining using Congo red or ThS did not show the presence of amyloid deposits (Fig. 4b). Amyloid deposition in FDD-Tg mice is particularly strong in the cerebellum (Fig. 4e) but not in Knock In mice (Fig. 4b) and only FDD-Tg mice showed immunoreactivity using Ab 1700, specific for the ADan amyloid peptide [20] (Fig. 4f versus 4c). Immunoreactivity was not seen in non-transgenic littermates, FDD-Tg and Knock In mice using antibody 4G8 (not shown). These results indicated that the brain of Knock In mice maintained normal morphology of aged mice and were free of amyloid deposits at 18 months of age.

### Biochemical Analysis of BRI_2-ADan_ Expression

**Figure 5:**
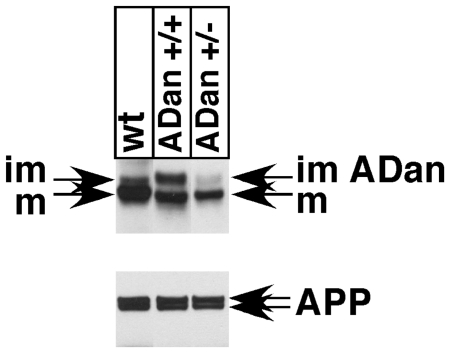
Biochemical analysis of BRI_2-ADan_. Lysates from E16-17 neuronal cultures of wt, ADan +/− and ADan +/+ mice were blotted with antibodies against BRI2 and APP. Upper panel shows impaired maturation of BRI_2-ADan_ compared to wild type; lower panel show normal pattern of expression and maturation of APP.

To determine whether the mutant proteins are expressed *in vivo*, we made protein extracts form neuronal cultures of wt, *BRI_2_^ADan/wt^* and *BRI_2_^ADan/ADan^* mice. Western blot analysis of these samples shows three phenomenona. 1) A common, major band of about 32 kDa is readily visible in all three samples. This band corresponds to the post-furin cleavage mature BRI_2_ (mBRI_2_) polypeptide because mBRI_2_ is identical regardless of whether it is derived from the wild type or the mutant precursor protein. 2) A much less abundant protein of ∼33 kDa which is visible in the wild type and *BRI_2_^ADan/wt^* sample but not the *BRI_2_^ADan/ADan^* lysate. This band corresponds to the wild type, immature BRI_2_ precursor (imBRI_2_). The absence of this band in the *BRI_2_^ADan/ADan^* neurons is attributable to the fact that these cells have both BRI_2_ alleles mutated. 3) As expected, the *BRI_2_^ADan/wt^* sample expresses all three BRI_2_ polypeptides, i.e. wild type imBRI_2_, BRI_2-ADan_ precursor and mBRI_2_. It is worth noting that, while immature murine BRI_2_ (imBRI_2_) is barely detectable, BRI_2-ADan_ is more markedly expressed (see *BRI_2_^ADan/ADan^* neurons, Figure 5) in this specific experiment. Whether this is related to the artificial absence of the wild type protein (this does not happen in FDD since the patience have only one mutated allele) or reflects some interesting biology of BRI_2-ADan_, remains to be determined. Notably, APP expression and maturation are comparable in FDD_KI_ and wt mice (Fig. 5, lower panel).

## Discussion

In this manuscript we present an initial characterization of a Knock In model for a human neurodegenerative disorder, FDD. This Knock In model is strategically different from the conventional genetic approach to cerebral amyloidosis. Traditionally, mouse models for human dementias are based on a transgenic approach in which human mutant proteins that cause familial forms of dementia are over-expressed under the control of brain-specific promoters [25], [26], [27]. This approach has both limitations and advantages. The potential limitations are linked to the fact that these models are genetically incongruous with the human diseases. These dementias have an autosomal dominant way of transmission and affected subjects have a wild type and a mutant allele. On the contrary, the commonly used animal models over-express, from an artificial promoter, several copies of a mini-gene coding the mutant protein. The mutant gene is therefore expressed in a spatio-temporal manner different from the natural alleles and in cells with two copies of wild type alleles. However, transgenic approaches have successfully reproduced cerebral amyloidosis in mice, and the reproduction of those lesions is one of the main parameters used to endorse an animal model as representative of the human disease. The opposite is true for a Knock In approach. In this genetic paradigm, the pathogenic human mutation is inserted in the mouse allele. Thus, the Knock In mouse is genetically faithful to the human pathology. However, the Knock In model may not successfully reproduce the amyloid lesion in the mouse. The model that we present here is a clear demonstration of the above-mentioned assumptions. A comparison of the FDD-KI model that we have created with the FDD transgenic model generated by Vidal et al [20] illustrates the superior power of a transgenic approach as far as the reproduction of amyloidogenic lesions are concerned.

In spite of this limitation, we believe that there is some merit to a Knock In approach. Human familial dementias may have a pathogenic component due to loss of function caused by the mutation [28], [29], [30], [31], [32]. This component may participate to the pathogenic process together with a gain of toxic function due to amyloidosis. A loss of function phenotype would be hidden by a transgenic approach for two reasons: 1) the two endogenous wild type alleles can support synthesis of sufficient amounts of functional wild type protein: 2) the over-expression of a partial loss of function mutant protein may augment rather then decrease that function, as may happen in the disease. It would be erroneous to assume that the absence of obvious neuropathological lesions (such as plaques, NFTs and/or neuronal cell loss) indicate absence of clinical pathology. Functional deficits may underlie the very first clinical manifestations of neurodegenerative diseases, and memory deficits may be caused not by gross anatomical changes but by subtle dysfunctions of the neuronal network. In this framework, determining the absence of neuropathological lesions in FDD_KI_ mice is intrinsically important. The behavioral characterization of this mouse model will test this hypothesis. In addition, the FDD_KI_ mouse will be useful to clarify the change of trafficking or processing of Danish mutant of BRI2 protein without potential artifacts due to over-expression in transgenic models. Finally, the FDD_KI_ mouse will be instrumental in studying the interaction between mutant BRI_2_ and APP *in vivo* and how the Danish mutation affects the inhibitory role of BRI_2_ on APP processing *in vivo*.
